# Adenine phosphoribosyltransferase deficiency as a rare cause of rapidly progressive chronic kidney disease

**DOI:** 10.1007/s40620-025-02304-7

**Published:** 2025-05-14

**Authors:** Hannes Alder, Ariana Gaspert, Daniel G. Fuster

**Affiliations:** 1 Department of Nephrology, Winterthur Cantonal Hospital, Winterthur, Switzerland; 2 Department of Pathology and Molecular Pathology, University Hospital Zurich, Zurich, Switzerland; 3 Department of Nephrology and Hypertension, Inselspital, Bern University Hospital, University of Bern, Bern, Switzerland

**Keywords:** Adenine phosphoribosyltransferase deficiency, Crystal nephropathy, Rare kidney disease, 2,8-dihydroxyadenine, Chronic kidney disease

## The case

A 67-year-old man with progressive loss of kidney function corresponding to an estimated glomerular filtration rate (eGFR) decline from 100 to 20 ml/min/1.73m^2^ within three and a half years was referred to our clinic. He reported feeling healthy. He was born to Tamil, non-consanguineous parents. His medical history was significant for well-controlled arterial hypertension and type 2 diabetes. He had a remote history of urinary infections during childhood but denied any renal colic, hematuria or passing of urinary stones. The patient's family history was unremarkable. He was treated with olmesartan, amlodipine, hydrochlorothiazide, and metformin.

On the first visit at our clinic, plasma creatinine was 266 μmol/L (3.0 mg/dL) corresponding to an eGFR of 21 ml/min/1.73m^2^ with a potassium level of 7.1 mmol/l and a bicarbonate level of 14 mmol/l. Urinalysis revealed an unremarkable urine sediment with a protein-to-creatinine-ratio of 257 mg/g and an albumin-to-creatinine-ratio of 81 mg/g. Ultrasound showed a coarse parenchymal calcification in the lower pole of the left kidney measuring 9 mm, but no kidney stones. Following the discontinuation of metformin, hydrochlorothiazide and olmesartan and the addition of sodium polystyrene sulfonate and sodium bicarbonate, plasma potassium normalized, yet kidney function only partially recovered to an eGFR of 31 ml/min/1.73m^2^.

**Fig. 1:**
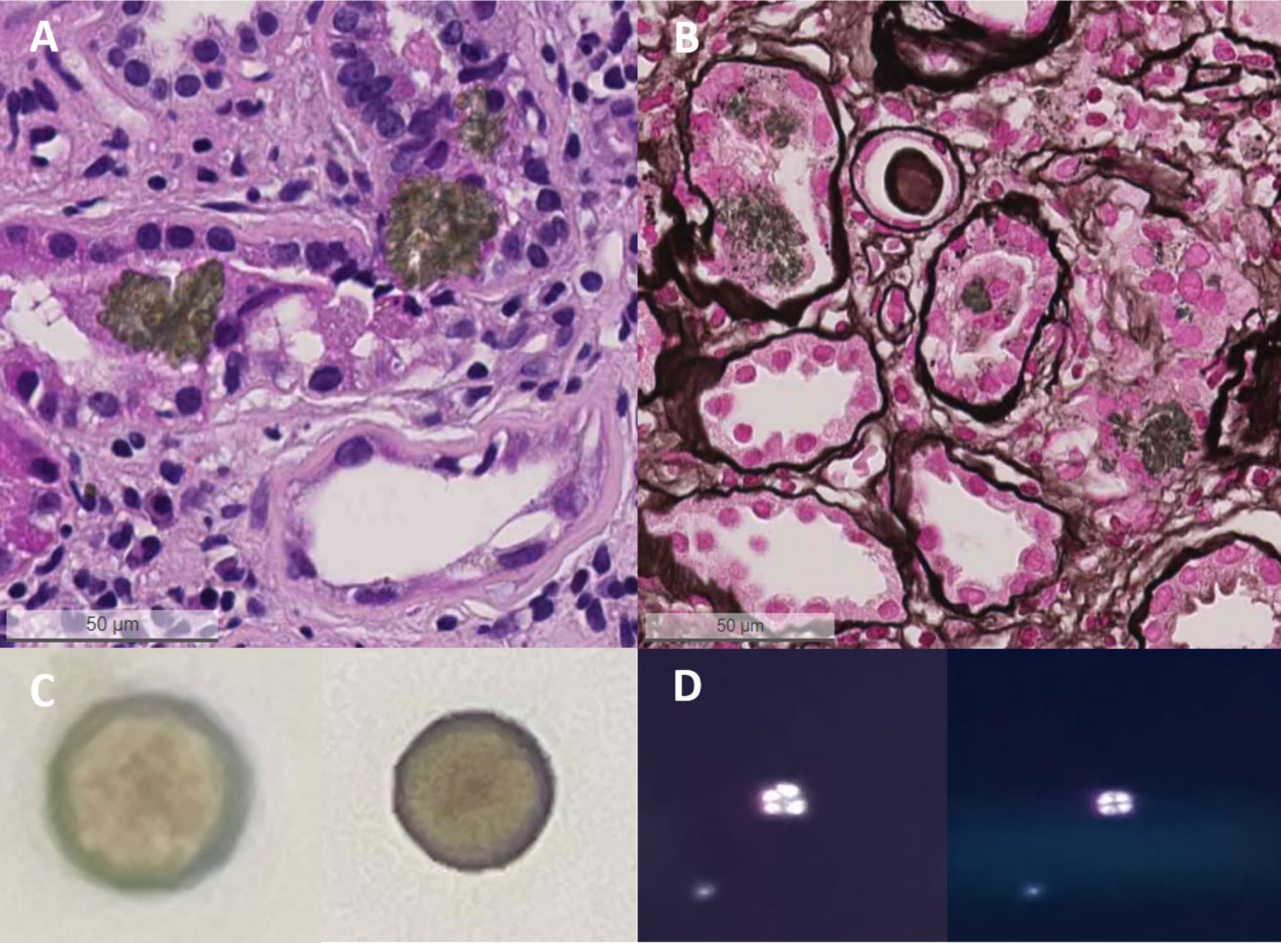
Kidney biopsy and urine microscopy showing dihydroxyadenine (DHA) crystals. **A** Brownish-green intratubular crystals in the Hematoxylin and Eosin (H&E) stain. **B** Grayish-black intratubular and interstitial crystals in the silver methenamine stain. **C** Urine light microscopy, DHA crystals appear round and brown. **D** Urine polarized microscopy, DHA crystals with characteristic central Maltese cross pattern

The patient subsequently underwent a kidney biopsy, which showed numerous birefringent, predominantly tubular and focal interstitial crystals (shown in Fig. 1A and B). The crystals appeared brownish green in the Hematoxylin and Eosin (H&E) and Periodic acid-Schiff (PAS) stains, light blue in the Acid Fuchsin Orange G (AFOG) stain, and grayish black in the silver stain, suggesting 2,8-dihydroxyadenine (DHA) crystals, which are pathognomonic for adenine phosphoribosyltransferase (APRT) deficiency. Another thorough microscopic urine examination was conducted and round, reddish brown crystals with a central Maltese cross pattern were identified (shown in Fig. 1C and D), confirming dihydroxyadenine crystalluria. The diagnosis of adenine phosphoribosyltransferase deficiency was confirmed by genetic testing. A homozygous pathogenic variant in the *APRT* gene (c.200G > A; p.Arg67Gln) was identified, which had been reported previously in patients with adenine phosphoribosyltransferase deficiency [1].

Allopurinol was initiated and then switched to febuxostat 80 mg daily. This led to a complete disappearance of dihydroxyadenine crystalluria and a continuous improvement in eGFR to 50 ml/min/1.73m^2^ over a period of 10 months.

### Lessons for the clinical nephrologist

Adenine phosphoribosyltransferase deficiency, also known as 2,8-dihydroxyadeninuria, is a rare and underdiagnosed autosomal-recessive disorder causing kidney stones and chronic kidney disease (CKD). Also in our case, the diagnosis was initially missed, likely due to lack of disease awareness and unspecific clinical presentation. However, its pathognomonic crystals were recognized on kidney biopsy. Adenine phosphoribosyltransferase is an enzyme that recycles adenine into soluble adenosine monophosphate. With adenine phosphoribosyltransferase deficiency, adenine is metabolized to dihydroxyadenine by xanthine oxidase (XO) instead. Dihydroxyadenine is excreted by the kidney but is highly insoluble, resulting in crystal precipitation in the renal parenchyma and the formation of kidney stones [2]. The frequency of asymptomatic heterozygosity for adenine phosphoribosyltransferase deficiency was estimated to be around 1%, which contrasts with the limited number of cases described in the literature. Adenine phosphoribosyltransferase deficiency can manifest at any age, but in ~ 50% of individuals symptoms do not occur until adulthood [2, 3]. Decreased kidney function was observed in about one third of patients at the time of diagnosis [2].

The diagnosis of adenine phosphoribosyltransferase deficiency is primarily based on the detection of dihydroxyadenine in stone composition analysis or the identification of dihydroxyadenine crystals in urine sediment or in a kidney biopsy. Urine microscopy is a sensitive, specific, and noninvasive tool for the identification of dihydroxyadenine crystals. Dihydroxyadenine crystals appear as round and brown, with a characteristic central Maltese cross pattern under polarized light [2]. Infrared spectroscopy should be used to identify dihydroxyadenine stones [4]. Since the identification of dihydroxyadenine stones or crystals is pathognomonic for adenine phosphoribosyltransferase deficiency, measurement of adenine phosphoribosyltransferase activity in erythrocytes, genetic testing or quantification of dihydroxyadenine in urine are not mandatory for diagnosis [4].

The cornerstone of treatment is high fluid intake, low purine diet and lifelong xanthine oxidase inhibition to prevent dihydroxyadenine crystal formation. Treatment with allopurinol has been shown to prevent kidney failure and even improve kidney function [2]. Edvardsson et al. demonstrated that febuxostat (80 mg/day) was more effective in reducing urinary dihydroxyadenine than allopurinol (400 mg/day) [5]. Family screening and genetic counseling should be offered since approximately 15% of individuals with adenine phosphoribosyltransferase deficiency may be asymptomatic [2; 3].

In conclusion, this case emphasizes the importance of raising awareness of adenine phosphoribosyltransferase deficiency as a rare cause of CKD. Adenine phosphoribosyltransferase deficiency can be diagnosed easily by urine microscopy and treated effectively with xanthine oxidase inhibitors, resulting in substantial recovery of kidney function.
